# Monthly Observation of the Number of Patients with Kawasaki Disease and its Incidence Rates in Japan: Chronological and Geographical Observation from Nationwide Surveys

**DOI:** 10.2188/jea.JE2008030

**Published:** 2008-12-17

**Authors:** Yosikazu Nakamura, Mayumi Yashiro, Ritei Uehara, Izumi Oki, Makoto Watanabe, Hiroshi Yanagawa

**Affiliations:** 1 Department of Public Health, Jichi Medical University, Shimotsuke, Tochigi, Japan.; 2 Epidemiology Unit, Tochigi Cancer Center Research Institute, Utsunomiya, Tochigi, Japan.; 3 Department of Preventive Cardiology, National Cardiovascular Center, Suita, Osaka, Japan.

**Keywords:** Mucocutaneous Lymph Node Syndrome, Incidence Rate, Geographical Distribution, Temporal Distribution, Japan

## Abstract

**Background:**

Although the epidemiologic features of Kawasaki disease for Japan have been observed, recently, chronological and geographical observations of the number of patients and incidence rate of the disease have not been conducted using observation units smaller than the whole country.

**Methods:**

We used the recent 5 nationwide surveys (the 15th to 19th) of Kawasaki disease in Japan, which covered patients for 10 years, i.e., between 1997 and 2006. The monthly number of patients by prefecture was calculated, and the number was corrected by the response rate of the target institutes of the prefecture. Chronological changes in the number of patients were observed by district. Geographical changes in the incidence rates were observed bimonthly by prefecture for the recent 6 years, from 2001 through 2006.

**Results:**

On the whole, the monthly number of patients increased gradually. For each year, the number was the highest in January and the lowest in October; it was relatively high during summer as well. Some differences existed among the districts with regard to the monthly observations. Bimonthly observations of the incidence rate by prefecture revealed the differences in the characteristics of the epidemics in different geographic areas.

**Conclusion:**

The chronological and geographical changes in the occurrence of Kawasaki disease in Japan for the recent 10 years suggested the involvement of one or more infectious agents in the occurrence of the disease.

## INTRODUCTION

Kawasaki disease is a syndrome that affects mainly infants and toddlers, and it causes systemic vasculitis.^1^^,^^2^ Even though the cumulative number of patients afflicted by Kawasaki disease amounts to more than 200 thousands since its prevalence in Japan in the 1960s,^3^ the etiology of this disease is still unknown. However, its epidemiologic features, such as 3 nationwide epidemics in 1979, 1982, and 1986, case reports of temporal clustering in small areas, and age-specific incidence rates, suggest an association between the disease onset and the presence of one or more infectious agents,^4^^,^^5^ which have not been identified.

If Kawasaki disease is related to the presence of infectious agents, the incidence of this disease may show geographic and temporal clustering, similar to other infectious diseases. Several studies have been conducted^6^^-^^11^ from this viewpoint, but there has been no recent nationwide study. In this study, using data from the recent 5 nationwide surveys of the disease in Japan, we observed the monthly occurrence of this disease for 10 years by district, and bimonthly occurrence for 6 years by prefecture.

## METHODS

In this study, we used the recent 5 nationwide surveys (the 15th to the 19th) of the Kawasaki disease in Japan.^3^^,^^12^^-^^14^ These 5 surveys covered patients with Kawasaki disease for 10 years, i.e., between 1997 and 2006.

**Figure 1:**
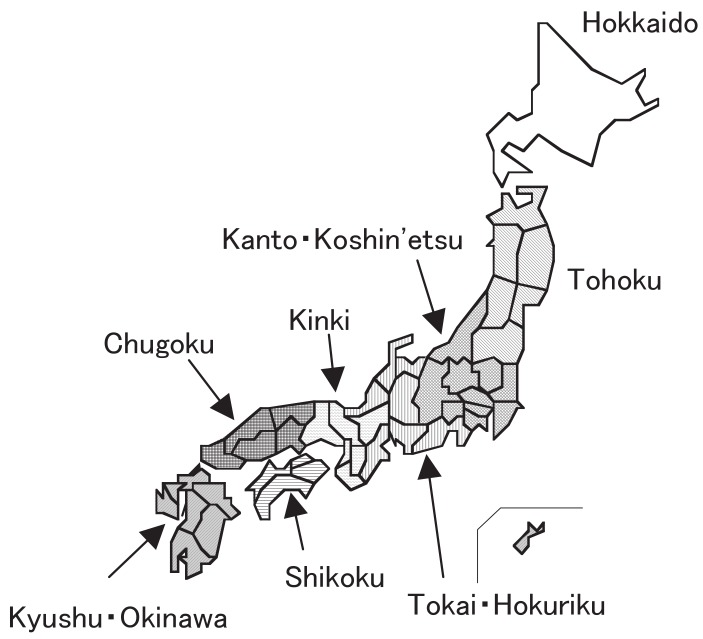
Location of districts in the study

The monthly number of patients with this disease was calculated by prefecture, and the number was corrected by the response rate of the target institutes of the prefecture. Because the response rates were distributed between 40% (Tottori in the 17th survey [2001-2002] and Tokushima in the 19th survey [2005-2006]) and 91% (Shimane in the 18th survey [2003-2004]), the monthly number reported to the surveys was divided by the corresponding response rates (by prefecture and survey), and these corrected numbers were analyzed. Chronological changes in the number of patients were observed in the 8 districts shown in Figure 1 as well as throughout Japan: Hokkaido, Tohoku (Aomori, Iwate, Miyagi, Akita, Yamagata, and Fukushima), Kanto-Koshin’etsu (Ibaraki, Tochigi, Gunma, Saitama, Chiba, Tokyo, Kanagawa, Niigata, Yamanashi, and Nagano), Tokai-Hokuriku (Toyama, Ishikawa, Fukui, Gifu, Shizuoka, Aichi, and Mie), Kinki (Shiga, Kyoto, Osaka, Hyogo, Nara, and Wakayama), Chugoku (Tottori, Shimane, Okayama, Hiroshima, and Yamaguchi), Shikoku (Tokushima, Kagawa, Ehime, and Kochi), and Kyushu-Okinawa (Fukuoka, Saga, Nagasaki, Kumamoto, Oita, Miyazaki, Kagoshima, and Okinawa).

Geographical changes in the incidence rates were observed bimonthly by prefecture because the rates were unstable for monthly observation due to the small population in some prefectures. The focus of the observation was on the rates in the recent 6 years, i.e., from 2001 through 2006. As is the international practice with Kawasaki disease epidemiology, the incidence rate of Kawasaki disease is expressed by the total number of patients (not only those aged 0-4 years) with the disease divided by the population aged 0-4 years.

## RESULTS

**Figure 2:**
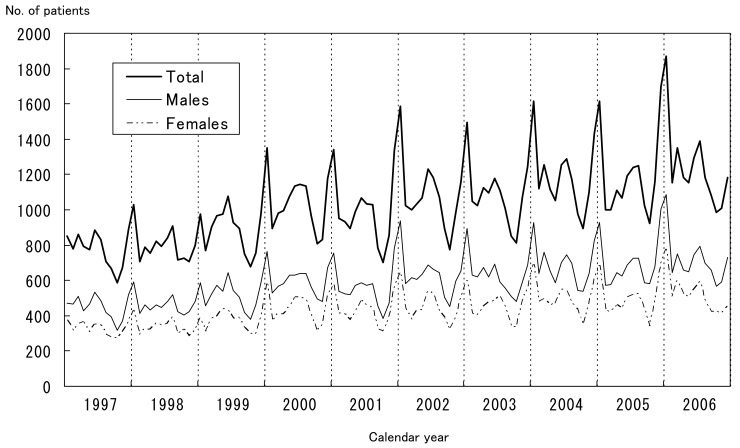
Monthly number of patients with Kawasaki disease (adjusted for the response rate to the survey) in Japan by sex, 1997-2006

Figure 2 shows the chronological changes in the monthly numbers of patients with Kawasaki disease in Japan by sex. On the whole, the monthly number of patients increased gradually. For each year, the number was the highest in January and the lowest in October, and it was relatively high during summer as well. The same tendency was observed in both sexes.

**Figure 3:**
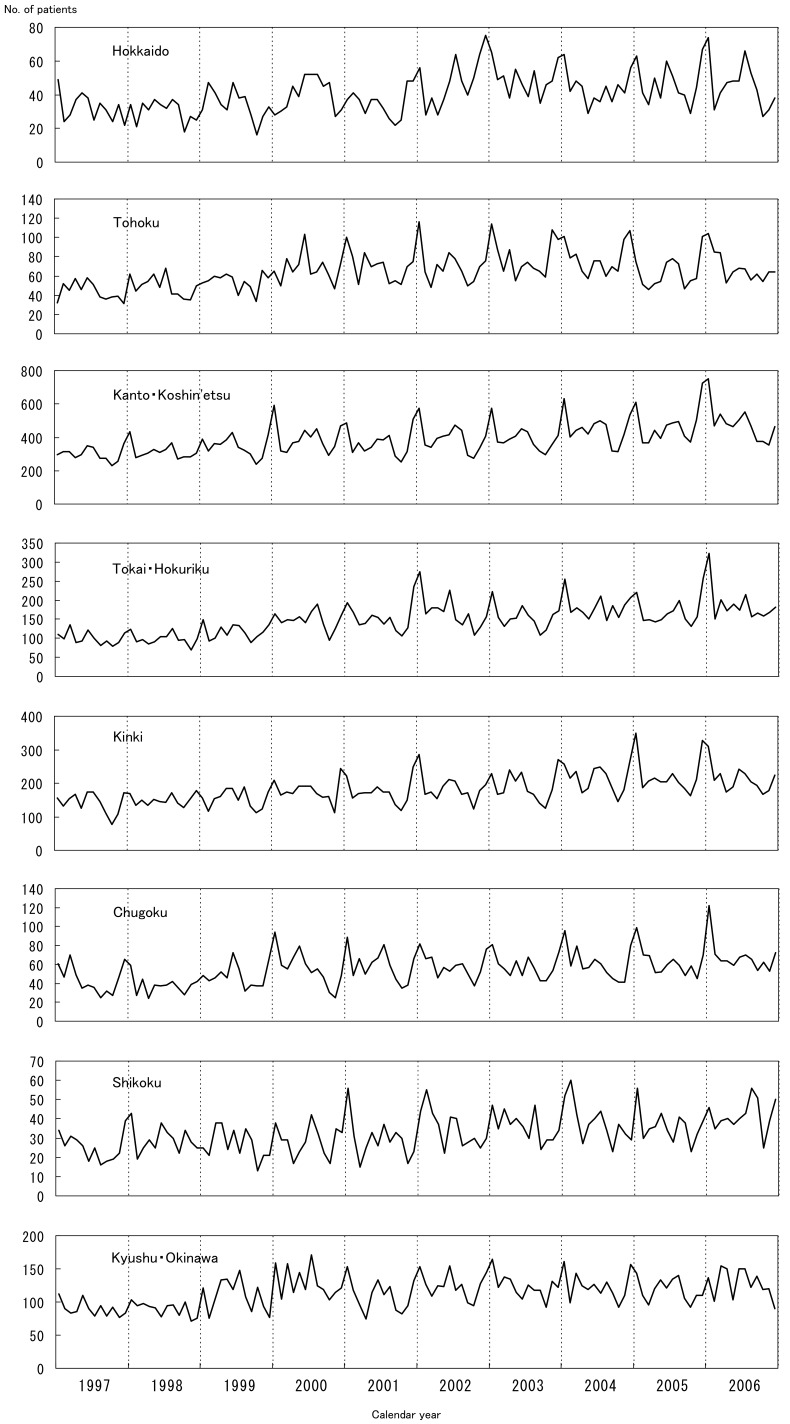
Monthly number of patients with Kawasaki disease (adjusted for the response rate to the survey) in Japan by district, 1997-2006

The results of the chronological observation by district are shown in Figure 3: there were some differences among the districts. In Hokkaido, the peak incidence recorded in January, which was observed throughout Japan, was not marked before 2002. In Tohoku district, the January peak was not manifested before 2001. Thereafter, the January peak was observed every year, but the summer peak was only observed in certain years (2002 and 2005). In Kanto-Koshin’etsu district, the January peak was obvious for all years except 1999, and the summer peak was obvious for almost all the years as well. The height of the January peak increased with each succeeding year in this district. Several manifest peaks (June 2002, July 2004, and August 2005) were marked in Tokai-Hokuriku district. In Kinki district, the peak in January 2003 was not as sharp as that observed throughout the country. The January peaks were not sharp in Chugoku district as well. In Shikoku and Kyushu-Okinawa districts, numerous spikes were observed on the epidemic curves. In summary, for the 8 districts, different characteristics were observed on the chronological epidemic curves, and none of them were identical to the epidemic curve for Japan as a whole, as shown in Figure 2.

**Figure 4:**
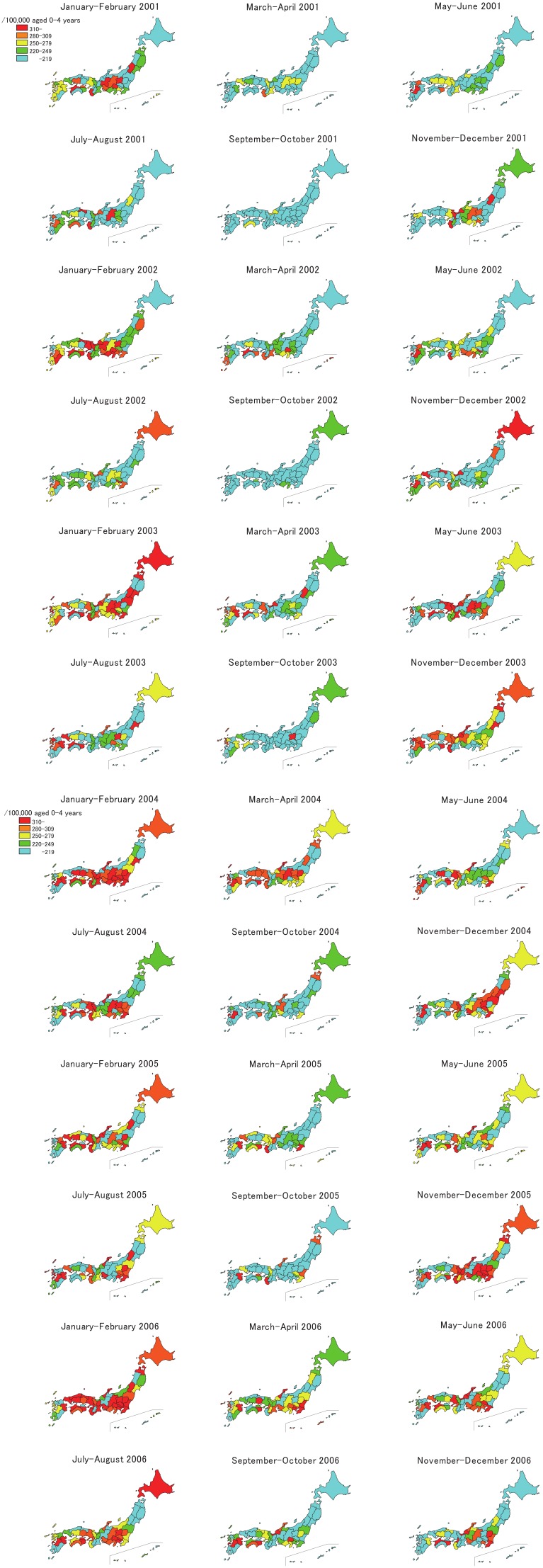
Bimonthly incidence rate (/100,000 aged 0-4 years/year adjusted for the response rate to the survey) of Kawasaki disease by prefecture in Japan, 2001-2006

The bimonthly incidence rates between 2001 and 2006 by prefecture are shown in Figure 4. For the first 2 months of the observation, the prefectures with high incidence rates were clustered in the central parts of the country. In the summer of this year, several prefectures, such as Nagano, Wakayama, and Tottori, showed high incidence rates. In the winter of 2001-2002, prefectural epidemics began in several parts of Japan, and the epidemics progressed to the prefectures in the central and then to the prefectures in the western part of the country. In 2002, high incidence rates were observed in some prefectures, such as Wakayama and Kumamoto. In the first 2 months of 2003, prefectures throughout the country had high incidence rates, which were different from the winter epidemic of the previous year. In the summer of 2003, several prefectures showed high incidence rates, and many prefectures showed high rates in November-December this year. The prefectures with high incidence rates were clustered in the Kanto, Chubu, Kinki, and Setouchi areas in the first 2 months of 2004. Following this, some prefectures showed high rates this year, and in November-December, the epidemics were observed mainly in the eastern part of Japan. However, in the next 2 periods that were observed, i.e., January-February and March-April 2005, the prefectures with high incidence rates were observed in the western part of the country. In July-August 2005, prefectures in Kanto area, such as Tochigi, Chiba, and Kanagawa, showed high incidence rates. At the end of this year, prefectures with high incidence rates were clustered mainly in the Kanto and Kinki areas. In the first observation period in 2006, many prefectures showed a high incidence rate, but many prefectures in Kyushu district and some in Tohoku district showed low incidence rates. In the summer of this year, epidemics were observed in the Chubu area and some prefectures in Shikoku and Kyushu. As shown above, observation by prefecture revealed differences in the characteristics of the epidemics in different geographic areas.

## DISCUSSION

In this study, we showed both temporal and geographic clustering of Kawasaki disease. In other words, it did not occur at random in time and space. In addition, the seasonal patterns of the occurrence slightly differed among districts.

As mentioned above, epidemiologic data have suggested that one or more infectious agents might be involved in the occurrence of this disease.^4^^,^^5^ If this is the case, the seasonal patterns shown in Figures 2 and 3 and the geographical shifting of the small epidemics shown in Figure 4 are reasonable. In addition, the high incidence rates in winters and summers might indicate that 2 or more infectious agents trigger the disease in different seasons.^15^ Figure 3 shows that the winter peak was sharper in the eastern part of the country than in the western part. If the agent types differed in both parts of Japan, this phenomenon was reasonable. However, no similar epidemic curve was observed for other well-known infectious diseases, which mainly affected children in the 1980s.^6^

As shown in Figure 4, all the prefectures in Japan had one or more periods of time with high incidence rates of Kawasaki disease. This feature was different from that of other noncommunicable diseases, such as cerebrovascular diseases, the mortality rates of which were observed to be constantly high in certain prefectures for a medium-term duration (for example, 10 years). From this viewpoint, one or more infectious agents are suspected to be associated with the occurrence of this disease as well. High incidence rates were observed in several prefectures despite the low rates in the surrounding prefectures, such as during May-June 2001 in Kumamoto, between July and October 2003 in Gunma, and September-October 2005 in Wakayama. Observation of the geographic distributions by municipality (city, town, and village) might provide some clues regarding the etiology of the disease; this will be conducted next as a part of the epidemiologic observation.

There were several limitations in this study. Because the response rates to the surveys were not 100%, the number of patients and incidence rates were corrected using the response rates. However, these values might be overestimated because the survey covered 80-90% of patients.^16^ The response rates were approximately 70%, and some of hospitals did not return the questionnaires because they did not have patients with Kawasaki disease. However, no other logical correction of the response rate existed, so we used this method. Although we have all the data of the past 19 nationwide surveys of Kawasaki disease in Japan, we used only data for the recent 10 years for the chronological observation, and data for 6 years for the geographical observation. The incidence rate of Kawasaki disease in Japan has increased gradually since the mid-1990s so that the focus of the chronological change observation was on the recent 10 years. The 6-year observation for the geographical pattern was limited for the recent 6 years because the seasonal patterns became manifest since 2001 throughout Japan, as shown in Figure 2.

In conclusion, we showed the chronological and geographical changes in the occurrence of Kawasaki disease in Japan for the recent 10 years, and the epidemiologic features suggested the involvement of one or more infectious agents in the occurrence of the disease.
